# Comparison of Overall Fit of Milled and Laser-Sintered CAD/CAM Crown Copings

**DOI:** 10.1155/2019/7310175

**Published:** 2019-07-07

**Authors:** Lucie Zuskova, Noor A. Al Mortadi, Robert J. Williams, Karem H. Alzoubi, Omar F. Khabour

**Affiliations:** ^1^Dental Hospital, University Hospital of Wales, Heath Park, Cardiff, CF14 4XW, UK; ^2^Department of Applied Dental Sciences, Jordan University of Science and Technology, Faculty of Applied Medical Sciences, Irbid, Jordan; ^3^Department of Clinical Pharmacy, Jordan University of Science and Technology, Irbid, Jordan; ^4^Department of Medical Laboratory Sciences, Jordan University of Science and Technology, Irbid, Jordan

## Abstract

**Background and Aims:**

The aim of this study was to investigate the effect of computer-aided design/computer-aided manufacturing (CAD/CAM) procedures on the overall fit of metal copings.

**Materials and Methods:**

A standardized die was made in die stone of an upper right molar prepared for a full crown. The die was digitalized by an Identica Blue Light Scanner, and the coping substructure was designed using CAD software. Ten milled specimens and ten laser-sintered specimens were manufactured by Renishaw plc based on the generated file by the software. All twenty copings were digitized by the Identica scanner, and the data were superimposed with the original premanufacturing data file of the prepared full crown. Using the Geometric Modelling Library (GML) package, the fit discrepancies were displayed as colour maps showing discrepancies in three dimensions. Each map was made up of thousands of data points carrying numerical error values allowing detailed analyses.

**Results:**

The milled group displayed a mean of fit discrepancies of 42.20 *μ*m (SD 3.04 *μ*m), while the laser-sintered group showed a mean of 42.24 *μ*m fit discrepancies (SD 2.94 *μ*m). Thus, a small difference of 0.04 *μ*m between the two groups was detected.

**Conclusions:**

The evaluated manufacturing systems can be used in dental practice as a small and insignificant discrepancy of fit between the two manufacturing methods was detected.

## 1. Introduction

Ceramometal crowns are the most common type of restoration used in dentistry [1]. Recently, types of restoration are commonly produced by various -aided design/computer-aided manufacture (CAD/CAM) systems rather than the conventional lost-wax technique introduced in 1907 by Taggart [2]. The advantages of using digital systems include the introduction of new and improved materials, reduced labor and time, increased cost efficiency, and more uniform, high quality [3]. The term “CAD/CAM” has come to be mostly associated with milling [2]. However, there are two possible ways of computer-assisted manufacturing (CAM): computer numerical control milling (CNCM) and direct metal laser sintering (DMLS) [4]. Both methods may use identical CAD technology to scan and design the restoration.

The milling procedure is a process in which a special cutter uses frequent abrasion to create shapes from a block of material [5]. The milling processes vary according to the number of milling axes from simple to more complicated. A greater number of milling axes facilitate milling of more complex geometries [2]. The milling devices use rounded cutters, the smallest diameter being 1 mm with most systems. Therefore, sharp and thinly extending edges and corners smaller than 1 mm would be impossible to mill. In order to overcome this, the so-called “ballooning effect” [2, 6] has been developed, which requires removing more material than would be expected by an alternative manufacturing method allowing a closer fit in angles. This expected discrepancy of fit is caused by over sizing of areas which are inaccessible for the milling head cutter to reach [2, 5, 7]. Therefore, it is essential for clinicians to adopt working procedures for preparations to help overcome the above problems, avoiding sharp angles and corners [2]. In spite of the expectations in relation to ballooning, there are many studies demonstrating impressive results for restorations manufactured by CNCM [8–11].

Direct metal laser sintering also called “3D printing” is a relatively new technique [12]. It is a process of building up dental frameworks by a high-powered laser beam focusing onto a bed of the Co-Cr alloy powder and welding it together into subsequent, thin solid layers on cooling [13]. The principle of this CAD/AM technology is in successive layering of alloy powder, creating layers about 0.020 mm thick [10, 14]. Potentially, complex dental devices can be produced and there could be an expectation that this method may be superior to milling due to the ability to fabricate complex angular structures which are difficult or impossible with subtractive (machining) technologies [15]. Furthermore, the waste materials after milling cannot be reused, making subtractive manufacturing material costs high. AM is emerging as a potential solution to the supposed problems of subtractive manufacturing because AM enables the creation of sophisticated geometrics and reduces manufacturing material costs [16]. The main advantage mentioned in most of the studies is the cost effectiveness of AM due to the nature of the technique and its minimal waste [12, 13]. A study [14] also commented that the precision of DMLS is revolutionary with the possibility of creating complicated shapes and geometries with thin sections in a range from 0.02 to 0.03 mm such as required for orthodontics [17, 18], removable partial dentures, maxillofacial prosthesis [19, 20], and intraoral sleep apnoea devices [21]. In addition, many other studies suggested this technique showed great promise as an alternative to the conventional casting technique [7, 12, 22, 23].

The precision of fit of a restoration is determined by two criteria: marginal fit providing a seal and an internal gap which should be uniform [1, 24]. Uniform internal fit allows for appropriate cement space important for good retention and resistance of the restoration [1].

It can be seen from the above that there has been little attempt to disaggregate the fit of CAD/CAM produced from conventionally produced restorations or further, to consider the different forms of CAM manufacture. This study seeks to address the latter imbalance. The aims of this study are to evaluate and compare the overall fit of metal copings fabricated by two different methods: CNCM (computer numerical controlled milling) and DMLS (direct metal laser sintering).

## 2. Materials and Methods

### 2.1. Master Die

One master model was selected with a typical 90-degree shoulder margin of an upper right molar full crown preparation. The preparation was chosen to represent common dental practice. The master cast was vacuum mixed and poured under vibration in Type IV die stone (Moonstone, Bracon Ltd., Etchingham, England) and sectioned and trimmed following a standard laboratory procedure.

### 2.2. Fabrication of Frameworks

Overall, 20 frameworks were fabricated with two different production techniques, providing ten specimens in each group. This was achieved by a single digitization of the master die by a noncontact Identica Blue Light dental Scanner (Renishaw plc). Calibration of the scanner was carried out prior to the scanning procedure. The design of the coping was undertaken in a CAD software package (exocad, Renishaw plc. gland) again using typical coping dimensions. The smart software automatically defined an ideal path of insertion and detected a marginal line. The thickness of the coping was set to 0.5 mm. According to the manufacturer's instructions, the parameters were set with a die space of 55 *μ*m starting 1 mm below the margin line. The master die was scanned and coping designed once using the same CAD data for both manufacturing groups in order to reduce variables. The CNCM specimens were manufactured by BEGO (Dental, Bremer Goldschlägerei Wilh. Herbst GmbH & Co. KG, Bremen, Germany) using Wirobond MI+ Co-Cr alloy blocks. DMLS frameworks were fabricated by an AM250 laser melting machine (Renishaw plc) using ASTM75 Co-Cr powder. The manufactured specimens were cleaned by grit blasting with 50 *μ*m aluminium oxide at a pressure of 5 bar prior to delivery. This also reduced reflectivity.

### 2.3. Digitization and Measurement of the Specimens

**Figure 1:**
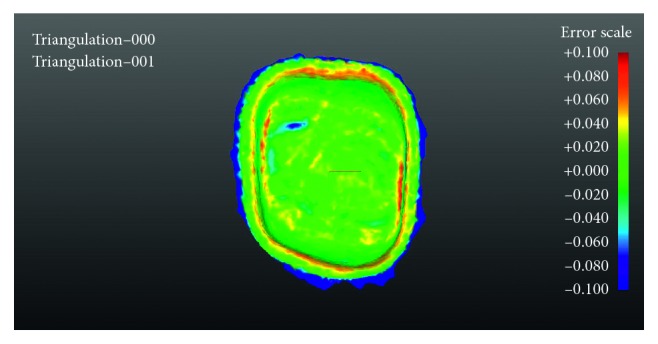
Computer numerical controlled milling 1 (CNCM1).

All copings were bedded occlusally into black scanning plasticine and sprayed on the fitting surfaces with white powder scanning spray (Proto3000, 3D engineering, Ontario). Following scanner calibration, all twenty specimens were digitized by the DS30 scanner (Renishaw plc) and filed according to their manufacturing group. Using the computer program package GML (Renishaw plc), the (stereolithography) STL file of the prepared tooth of master data were superimposed with each STL coping specimen files of the fitting surfaces. The program mathematically relocated each coping onto the master model by choosing the best object-to-object penetration, creating a graphic image. The images visually represented the differences in fit by colours (Figure 1). The colour scale showed discrepancies between −0.1 *μ*m and +0.1 *μ*m with green and blue shades indicating negative deviations whereas yellows and reds indicated positive deviations. Measurements were performed by extracting approximately 6000 data points from the whole area of the fitting surface of each specimen. Every data point represented a numerical value of a deviation in *μ*m and its specific location on the *XY* and *Z* axes.

### 2.4. Statistical Analysis

Statistical computations were made in Excel 2007 (Microsoft Office, Redmond, Washington, USA). Mean, standard deviation (SD), and standard error (SE) values were calculated using the program functions, and the data are recorded in Table 1. Groups were compared using Student's *t*-test.

## 3. Results

**Figure 2:**
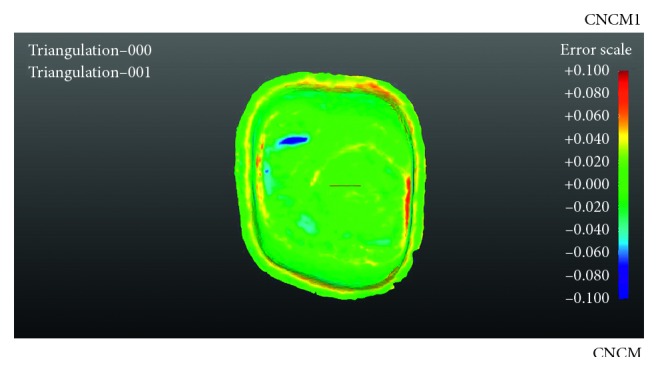
Computer numerical controlled milling 2 (CNCM2).

A general assessment of the aligned colour-coded images (Figure 2) showed differences between the two groups. The CNCM images did not indicate much deviation. There was a moderate degree of yellow and red colours signifying positive deviations on mesial and distal triangular fossa of the restoration and on the border of the spacer line close to the margin. Unexpectedly, DMLS images appeared to have a greater range of fit deviations to CNCM as indicated by the colour spectra. The occlusal aspect of the fitting surface of the restorations was the area mainly affected with blue colour patches, indicating negative deviations over the occlusal aspects underlying cusps while showing positive deviations mesiodistally. Also, marginal aspects of the copings showed some positive deviations.

The quantitative analysis of the data illustrated in Tables 2 and 3 show very similar measurements for both groups. On average, 6000 data points containing the error values were extracted for each specimen. To find the difference between the two groups, the mean of all data points for each coping was calculated together with the SD and SE. The smallest and largest deviation values found are also shown in the Tables under minimal and maximal value. Total mean of discrepancies for the CNCM group was found to be 42.20 *μ*m with an SD of 3.04 *μ*m. The DMLS group showed a mean discrepancy of 42.24 *μ*m with an SD of 2.94 *μ*m. Although, the CNCM group showed a slightly lower value for the mean total with a difference of 0.04 *μ*m, the DMLS group was shown to have less variation from the average than the CNCM group. Both groups revealed very low SDs that indicate the data points were very close to the mean. Furthermore, the small SEs also indicated the mean was close to the true mean of the groups.

## 4. Discussion

The findings of this study cannot be used to draw definite conclusions without consideration of the nature of the study and chosen methodological approach. However, interesting information about overall fit of Co/Cr copings fabricated by two different digital manufacturing methods was found.

Although, the results of this study rejected the expectation arising from the literature review that a difference would be found between two groups, the actual difference was fractional and, again unexpectedly, in favour of the CNCM group. When the findings from the two manufacturing groups were compared, on average, CNCM specimens were shown to have a slightly better fit than the DMLS group. However, the difference of 0.04 *μ*m could be described as negligible. Interestingly, both groups also showed very good consistency between the specimens with minimal variation from the mean.

Comparisons of findings with previously conducted research can be difficult because of the differences in the methodological approaches. A study [25] presented the mean marginal gap of DMLS copings as low as 75 *μ*m and an internal gap of 99.8 *μ*m. On the contrary, a study [11] found a marginal fit of 102.09 *μ*m and an internal fit of 268 *μ*m. These are very contrasting findings which do not compare to the findings of a mean discrepancy of 42.24 *μ*m for the DMLS group observed by this study. A similar study completed [7] also investigated the comparison of fit between groups of CNCM and DMLS frameworks. The difference in methodology should be considered as the findings showed the DMLS group had the best fit with a significant difference (*p* < 0.05) based on the mean of discrepancies of 84 *μ*m and 166 *μ*m for CNCM frameworks. The only study with a similar methodological approach was undertaken [5] which observed a mean discrepancy of internal fit of CNCM onlays of 38 *μ*m. These results are very comparable to the mean of discrepancy 42.20 *μ*m for the CNCM group in this study. This could be due to the three-dimensional data analysis protocol used in both studies.

Although both methods of measuring fit are claimed to have high reliability by the authors mentioned above, more limitations could be seen by choosing a small number of locations for measurements in the same plane which are prone to variation. The studies mentioned above used between 4 and 11 measuring locations for each restoration. By contrast, the current study used approximately 6000 data points per restoration. It is clear that the advanced digital measuring method allowed for a comprehensive and more precise analysis.

Previous studies have taken various approaches in creating master dies and specimen dies. Some investigators chose stainless steel as a material for master dies [23, 24]. Others used brass [6, 26] but the majority used dental stone [5, 11, 26, 27]. For this study, it was possible not to use duplicate dies to maintain consistency. Duplication could potentially create inaccuracies. Although die stone does not have such a high wear resistance as stainless steel, because of the method of scanning subjected the die to barely any pressure, the material was adequate for this study. Moreover, die stone is more easily scanned than stainless steel. Using the same die and also same CAD to create the digitally produced specimens was a way to reduce the number of variables.

Some studies have used nonanatomical preparations for their master dies such as regular conical shapes with round edges and flat tops [7]. However, these are not typical preparations. The current study was an attempt to make “in vitro” measurements more transferrable to the “in vivo” environment. Furthermore, the geometric shape of the die could influence the result.

Contrastingly, the DMLS image analyses were seen to have more distinctive colours showing negative and positive deviations. This could be explained as being due to surface roughness of the laser-sintered coping created during the manufacturing process [24]. A possible reason for a decrease in fit may be that the layering process produces steps [13]. The layer thickness for this study was 20 *μ*m, which is bigger than the scale set for the colour map analysis. Therefore, deviations could appear. Another possibility is that the layers were fused together by laser which could be expected to cause thermal expansion of the alloy.

## 5. Conclusion

Within the limitation of this study, it can be concluded that the difference between overall fit of CNCM and DMLS copings was negligible. In fact, both manufacturing techniques have shown excellent results in terms of overall fit and consistency. Consequently, either of the systems investigated would be highly recommended for use in dental practice depending on individual preferences.

## Figures and Tables

**Table 1:** Chemical composition of alloys as a weight percentage according to manufacturers.

	Co	Cr	Mo	W	Si	Fe	Mn
Wirobond® MI+	63.8	24.8	5.1	5.3	≤1	—	—
ASTM75	61.8–65.8	23.7–25.7	4.6–5.6	4.9–5.9	0.8–1.2	0.0–0.5	0.0–0.1

**Table 2:** Overall fit discrepancies of the CNCM group.

Specimen name	Mean	Minimal value	Maximal value	Standard deviation	Standard error (*μ*m)
CNCM1	42.34	37.21	47.17	2.905	0.04
CNCM2	42.27	36.65	47.72	2.871	0.04
CNCM3	42.17	35.95	48.13	3.254	0.04
CNCM4	42.22	36.64	47.61	2.912	0.04
CNCM5	42.35	36.56	48.02	3.161	0.03
CNCM6	42.12	36.15	47.68	2.927	0.05
CNCM7	42.15	35.86	47.65	2.915	0.05
CNCM8	41.93	35.72	48.03	3.204	0.03
CNCM9	42.17	36.68	47.58	2.882	0.04
CNCM10	42.27	36.01	48.33	3.323	0.04
Mean total	42.20	36.34	47.79	3.04	0.04

**Table 3:** Overall fit discrepancies of the DLMS group.

Specimen name	Mean	Minimal value	Maximal value	Standard deviation	Standard error (*μ*m)
DLMS1	42.27	36.93	47.49	2.862	0.04
DLMS2	42.25	36.79	47.49	2.908	0.04
DLMS3	42.12	36.48	47.40	3.019	0.04
DLMS4	42.22	36.48	47.46	2.933	0.04
DLMS5	42.36	36.81	47.85	3.026	0.04
DLMS6	42.23	36.80	47.49	2.887	0.04
DLMS7	42.17	37.35	47.12	2.834	0.04
DLMS8	42.25	37.03	47.41	2.914	0.03
DLMS9	42.40	36.71	47.89	3.040	0.04
DLMS10	42.14	36.42	48.45	2.956	0.04
Mean total	42.24	36.78	47.50	2.94	0.04

## Data Availability

The data used to support the findings of this study are available from the corresponding author upon request.
